# The effects of simulation-based education on medical students' motivation

**DOI:** 10.5116/ijme.60c0.981e

**Published:** 2021-06-29

**Authors:** Parisa Moll-Khosrawi, Christian Zöllner, Jonathan S. Cronje, Leonie Schulte-Uentrop

**Affiliations:** 1 Department of Anaesthesiology, University Medical Center Hamburg-Eppendorf, Martinistr. 52, 20246 Hamburg, Germany

**Keywords:** Learning, motivation, simulation-based medical education, teaching approaches, construction of medical curricula

## Abstract

**Objectives:**

To assess the effects of simulation-based education on medical students' motivation and to compare these effects with the motivational effects of a classical teaching approach (seminar).

**Methods:**

In this cross-sectional study, motivational qualities of 164 3rd year medical students, who participated in four mandatory simulation-based training and two seminars of the department of anaesthesiology, were assessed. Comparative analysis was made to determine differences and changes of motivation towards participating in each teaching unit and each teaching format, using a one-way analysis of variance and unpaired t-tests.

**Results:**

The different motivational qualities, as well as the computed levels of autonomous and controlled motivation of students towards participating in each of the six teaching units and each teaching format did not differ significantly (F _(5, 839) _= 0.66, p = 0.657; F _(5, 839)_ = 0.29, p = 0.920; (t _(843)_ = - 0.72, p = 0.471; t _(843) _= -0.17, p = 0.868). Students` motivation, particularly autonomous motivation, did not enhance after participating in the first SBME, (t _(264)_ = 1.035, p = 0.301), after participating in the second SBME, (t _(254)_ = -0.055, p = 0.956), or after participating in the third training (t _(250) _= -0.881, p = 0.379).

**Conclusions:**

Simulation-based medical education provides a valuable teaching approach but, in this study, this teaching approach did not enhance nor stimulate student motivation. Therefore, simulation-based medical education equals classical teaching approaches regarding student motivation. Further investigations are needed to identify how simulation-based medical education could enhance medical students' motivation.

## Introduction

Medical undergraduate curricula have changed and grown in the past years to provide the best learning environment for students. These changes were mostly based on content concerns (what to learn) and dealt with transmission and procession (how to learn) of knowledge.^1^^,^^2^ According to educational psychology, learning can be mapped on three dimensions, and all these dimensions have to be considered to create learner-oriented teaching and truly student-centred curricula.^3^^-^^5^ However, recent curriculum changes only aimed at two dimensions of learning, namely the cognitive and metacognitive.^5^  The affective (motivational) dimension has been neglected and under-evaluated.^6^

To understand the motivational dimension of learning and its impact on medical students, a brief insight into one of the leading theories of motivation, which has proven good applicability in medical education, is necessary.^7^^, ^^8^  The Self-determination theory (SDT), introduced by Deci and Ryan^9^ postulates that every human has an innate will to grow, which is hampered or supported by the satisfaction of the three basic psychological needs: Autonomy, competence and relatedness.^9^^,^^10^ The extent to which the basic psychological needs are satisfied, determine the manifestation of different types of motivation.

In SDT, motivation is qualified on a spectrum- one of its ends is intrinsic motivation (activities are carried out due to inherent satisfaction), and the other end is amotivation (lack of motivation).^11^^,^^12^ When activities are carried out on the basis of external sources, extrinsic motivation is present.^12^ Based on the levels of self-determination and autonomy, extrinsic motivation is subdivided into different forms of behavioural regulations: external-, introjected-, identified- and integrated regulation.^12^ These behavioural regulations differ in their levels of autonomy, in the following descending order: Integrated-, identified-, introjected- and extrinsic regulation.^13^^-^^15^

"Autonomous self-regulation" is the behavioural regulation which is desired and leads to better individual performance. It is composed of intrinsic motivation and integrated regulation, whereas "controlled self-regulation" is composed of external and introjected regulation.^15^^,^^16^ The integral relation of motivation and learning in an academic setting and for medical students has been compiled, identifying motivation as a determining factor for successful learning.^17^^,^^18^ It has been demonstrated that autonomous self-regulation leads to better learning and well-being^19^^, ^^20^ as well as greater time investments for studies^21^^-^^23^ less drop-out^20^ and academic success.^24^^-^^26^ Therefore, the goal of further curriculum developments should be the consideration of the motivational dimension of learning, as students' motivation might have a greater impact on individual outcomes than learning and teaching strategies.^7^ One step towards this goal is the identification of teaching approaches and formats that have stimulating effects on students' motivation.^27^

So far, evidence about the motivational effects of different teaching approaches and instructional designs is scarce. 5 One teaching approach which has the potential (based on SDT) to influence students' motivation and has already been implemented in many medical curricula is simulation-based medical education (SBME).^28^ Therefore, our study aimed to analyse the effects of SBME on student' motivation and to compare the motivational effects of SBME with those of classical teaching approaches. A cohort of 3rd-year medical students, who participated in a total of six SBME and classic seminars during the study period of one semester, was investigated. We aimed to assess the motivational levels of students towards participating in SBME and classical seminars and to explore changes of motivation after participation in each teaching format, especially after participation in SBME.

We hypothesised that students report higher levels of autonomous motivation towards participating in SBME than in the seminars and that autonomous motivation would increase after participation in SBME.

## Methods

### Study design and participants

We performed this cross-sectional study at the Department of Anaesthesiology in the University Medical Center of Hamburg-Eppendorf, Germany, during the winter semester 2018/19. The local Ethic Committee of Hamburg (Ethikkommission der Ärztekammer Hamburg, Hamburg, Germany) was contacted with a detailed project description, and the head of the committee rated the study with humans but not on humans and therefore did not see any necessity of deliberation and classified the project as not appropriate for ethic consultation (9 des Hamburgischen Kammergesetzes für Heilberufe). Third-year students aligned (medical undergraduate schedule of the curriculum) to participate in six compulsory anaesthesiology teaching units during the study period were eligible for the study (non-probability sampling). The 3rd year students were chosen because they were familiar with each teaching format from previous semesters. Hereby, cognitive bias due to unfamiliarity with the teaching unit and therefore bias of the motivational reports were ruled out.

One week prior to their scheduled teaching units, an email with a description of the study was send to the 3rd year students (N = 164). Participation was voluntary, and students were informed that no disadvantage would arise if they did not participate. We attained written informed consent from each study participant. All the 3rd year students of the study period (N = 164) participated in the study.

### Study setting and procedure

During the study period, the participants attended six compulsory teaching units of our department (one seminar on anaesthesiology, one seminar on pain medicine, three simulation-based emergency training on anaesthesia and cardiac arrest).

The students were divided into small groups by the student deanery, and all teaching units were attended in those small subgroups. For each small group, the six teaching units were scheduled within two weeks. Each undergraduate attended the teaching units in the same chronological order.  The seminars had predefined learning objectives which were accessible via an online platform of the faculty and were held like classic frontal teaching. Enough time for questions was provided, and interaction was encouraged. Each simulation training had a standardised set of scenarios, and each scenario was conducted by a group of three students. Enough time was provided for the students to practise skills and engage in the simulation scenarios. A systematic debriefing followed after each scenario, providing emotional support and adequate feedback.

### Data collection

We assessed situational motivation within the investigated group of students towards each teaching unit (repeated measures at six time points), using an adapted, translated and validated version of the Situational Motivation Scale (SIMS).^29^

Directly before each teaching unit, the written informed consents were collected and the paper-based SIMS questionnaire were handed out to the students. Enough time was provided to fill out the SIMS and the medical educators left the teaching room. After the teaching unit, the SIMS questionnaires and informed consents were collected by one person of our department, who carried out pseudonymisation of the data.

### Situational motivation scale (SIMS)

The SIMS measures the qualities of motivation that are present while engaging in an activity (specific point of time).^29^ It focuses on the important question of why an individual shows a specific behaviour.^30^ Therefore, it is possible to compare the motivational measurement with its conceptual definition that refers to the recognised reason of task engagement.^15^^,^^31^^,^^32^

To measure students' motivation, we asked them to specify the extent to which each item of the SIMS represented a reason for them to participate in the teaching unit and, specifically, the teaching format.

The adapted version of the SIMS^33^ measures intrinsic motivation, identified, introjected and extrinsic regulation as well as amotivation on four scales, each consisting of five subscales, resulting in twenty items. Each item has a 7-point Likert scale (1 ="Does not correspond at all" and 7 = "Corresponds exactly"), and each motivational quality has five categorised items.^33^  For example, to calculate the level of introjected regulation, the mean value of the sum of items number 5, 10, 15, 20 has to be computed (German version).^34^ A computed autonomous motivation index can be calculated by adding intrinsic motivation and identified regulation. Simultaneously, a computed controlled motivation index can be calculated by adding extrinsic and introjected regulation.  Validity and reliability of the SIMS, the adapted SIMS as well as the German translation have been reported.^29^^,^^33^ There are no specific cut-off values for the sub scales of the SIMS, describing if a type of motivation is too low. However, the scores can be interpreted in regard to differences.

### Data analysis

Statistical analysis was performed with IBM SPSS Statistics (version 23.0, IBM Corp., Armonk, New York, USA). We calculated the motivational indices (autonomous and controlled regulation) by adding the referring scales and applied descriptive statistics to calculate mean values, standard deviations and errors.

For the first hypothesis, we conducted two sub-analyses: For the first sub-analysis, we compared each motivational quality of each teaching unit separately. We identified differences in the quality of motivation towards participating in the teaching units (mean differences in situational motivation) and also the differences of motivation after and before each teaching unit, by conducting a one-way analysis of variance (ANOVA), after homogeneity of variances was asserted using Levene's test.

For the second sub-analysis, we compared the sum of each motivational quality for all the SBME teaching units with the sum of each motivational quality for all the seminars. The comparison of the sum of the scores was conducted, applying an unpaired T-test. Normality of distribution was assessed by the Shapiro-Wilk test.  The motivational qualities reported for each SBME were compared in the attended chronological order for the second hypothesis, conducting an unpaired t-test (comparison of first training with second, second with third and third with fourth).

## Results

A total of 981 (6 assessments per undergraduate for the same teaching units) assessments of situational motivation (SIMS) were collected. Fourteen of the SIMS questionnaires were incomplete and therefore excluded from the analysis.  Table 1 gives an overview of the number of assessed Situational Motivation Scale (SIMS) questionnaires collected at each teaching unit and the chronological order in which the teaching units were attended.

**Table 1:** Number of included SIMS questionnaires from each teaching unit of the 3rd year students

Order	Teaching unit	Number of included SIMS questionnaires
1	Seminar anaesthesiology	158
2	ACLS II	161
3	OR-Simulation	162
4	Seminar pain medicine	164
5	ACLS IIIa	162
6	ACLS IIIb	163

Note: 14 SIMS questionnaires were not complete and therefore excluded from the analysis. ACLS= Advanced Cardiac Life Support; OR= Operating room.

Motivational levels for all teaching units and the ANOVA results are shown in Table 2. The teaching units are depicted in the attended chronological order. Overall, the students reported high levels of intrinsic, identified- and autonomous regulation (motivation) for each SBME as well as for each seminar. Levels of external-, introjected- and controlled regulation as well as amotivation were reported low for each teaching unit (Table 2).

Our results did not confirm our hypothesis that students report higher levels of autonomous motivation towards participating in SBME than in the seminars.  We analysed the effects of each of the six teaching units on students' motivation separately (first sub-analysis) by conducting a one-way ANOVA. There were no outliners, according to the inspection of a boxplot, and the data was normally distributed for each group (Shapiro-Wilk test, p>0.059). Homogeneity of variance was given, as assessed by Levene's test, p > 0.05.  The levels of autonomous motivation for each attended teaching unit did not differ statistically, F_(__5, 839)_ = 0.66, p=0.657. Neither did the levels of the other reported motivational qualities (intrinsic, introjected, extrinsic, controlled, identified) differ significantly.

Our results indicate that the students had the same manifestations of motivation for participating in SBME or classical seminars. Previous seminars or SBME did not change the further levels of motivation: motivation did not increase nor decrease over the semester after attendance of each teaching unit, no matter which teaching units were compared (Table 2).

**Table 2:** Motivational qualities reported for each teaching unit and ANOVA results

Situational motivation	Seminar Anaesthesiology		ACLS II		OR-Simulation		Seminar pain medicine		ACLS IIIa		ACLS IIIb		ANOVA		
	M	SD	M	SD	M	SD	M	SD	M	SD	M	SD	F (df_N,_df_D_) F (5, 839)	p	η²
Intrinsic	5.86	.69	5.84	.72	5.86	.82	5.77	.83	5.81	. 78	5.90	.81	.52	.763	.00
Identified	5.39	1.0	5.49	1.0	5.25	1.2	5.29	1.2	5.31	1.2	5.43	1.1	.87	.502	.01
Introjected	2.73	1.1	2.77	1.2	2.78	1.2	2.81	1.3	2.81	1.3	2.85	1.4	.13	.987	.01
External	1.81	.85	1.85	.87	1.65	.78	1.77	1.0	1.80	.81	1.84	.96	.81	.543	.00
Amotivation	1.53	.70	1.52	.77	1.4	.78	1.50	.75	1.45	.74	1.40	.60	.53	.757	.00
Motivation Indices															
Autonomous	5.63	.75	5.66	.76	5.56	.92	5.53	.90	5.56	.89	5.66	.88	.66	.657	.00
Controlled	2.27	.85	2.31	.87	2.21	.86	2.30	.94	2.31	.89	2.34	.95	.29	.920	.00

Note: dfN indicates degrees of freedom numerator; dfD indicates degrees of freedom denominator; OR = Operation Room; ACLS= Advanced Cardiac Life Support

For the second sub-analysis (motivational qualities for all the SBME teaching units vs motivational qualities for all the seminars), the sum scores of the reported autonomous levels for all SBME and all seminars were calculated and compared.  The summed autonomous and controlled scores were comparable for both teaching formats (sum of SBME:  Mautonomous=5.61, SD=0.86; sum of seminar: Mautonomous=5.57; SD=0.85; sum of SBME: Mcontrolled =2.29, SD=0.89; sum of seminar: Mcontrolled=2.28, SD= 0.90).

There was no statistically difference between the sum of autonomous motivation reported for participation in all SBME or all classical seminars, t_(__843)_ =-0.72, p = 0.868. We also did not find any other significant differences for the other summed motivational qualities reported for all SBME and all seminars (Table 3).

**Table 3:** Summed means and differences of motivational qualities towards SBME and seminars

Motivational quality	SBME M	SD	Seminar M	SD	t (843)	p	95% CI	
							LL	UL
Intrinsic	5.85	.78	5.80	.78	-.83	.41	-.15	.06
Identified	5.37	1.15	5.33	1.23	-.58	.57	-.20	.11
Introjected	2.80	1.30	2.78	1.21	-.19	.85	-.19	.16
External	1.79	.86	1.78	.95	-.03	.98	-.13	.12
Amotivation	1.46	.73	1.51	.73	.89	.38	-.06	.15
Autonomous	5.61	.86	5.57	.85	-.72	.47	-.16	.07
Controlled	2.29	.89	2.28	.90	-.17	.87	-.13	.11

Note: SBME= Simulation-based medical education; CI = confidence interval; LL = Lower limit; UL = Upper limit.

**Figure 1:**
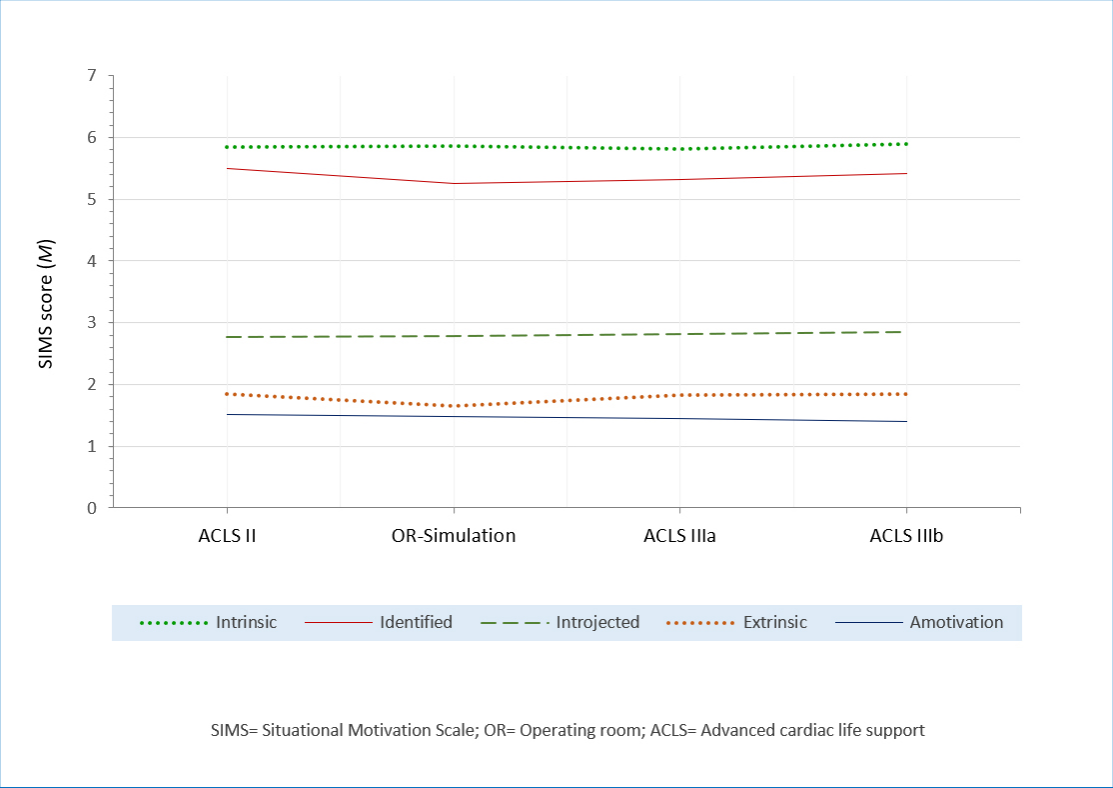
Development of 3rd-year students' situational motivation towards participating in the simulation-based teaching units of the study period

Figure 1 shows the chronological sequence of the students' motivation towards participating in the simulation training. We could not detect any effect of participating in SBME on student motivation during the study period: The reported motivation levels did not increase or decrease after each simulation training.

Our second hypothesis, that students´ motivation would increase after participation in SBME, was not confirmed by our results. Autonomous motivation did not enhance after participating in the first SBME, t_(__264)_ = 1.035, p = 0.301, after participating in the second SBME, t_(254)_ = -0.055, p = 0.956, or after the third training, t_(250)_ = -0.881, p = 0.379.

## Discussion

Our cross-sectional study found that 3rd-year medical student's motivation towards participating in simulation-based medical education (SBME) and classical seminars did not differ. Participation in SBME had no effect on students' motivation and did not enhance or decrease any motivational quality. Evidence about the effects of SBME on student motivation is scarce. Only a few studies pointed out that SBME is valued by students 35 and in postgraduate surgery training, simulation training lead to increased motivation for further training.^36^ Our hypotheses, that students' would have higher autonomous motivation towards participation in SBME than in classical seminars and that SBME would increase autonomous levels of student motivation, were based on the various benefits that SBME yields. SBME creates an ideal educational environment by bridging the gap between the classroom and clinical setting^37^ and hereby enhances student-directed learning.^38^^,^^39^Furthermore, SBME has the potential to increase autonomous motivation due to the specific perceived task value of training and perceived self-efficacy, as well as the feeling of competency during simulation training.^40^^,^^41^

However, although we put maximal effort to enhance students' autonomy during SBME by creating a good learning environment and providing appropriate feedback and emotional support^42^^, ^^43^ we could not find a modifying effect of SBME on students' motivation. As the investigated students were familiar with the teaching formats, potential bias due to the unfamiliarity and inquisitive effects on motivation, which new teaching formats may have^40^ can be ruled out. The students had not participated in too much simulation training before the study, and therefore, a certain state of boredom was prevented, which also could have had biased the results because boredom has shown to have a fading effect on intrinsic and identified regulation and thus autonomous motivation.^40^

One limitation of our study is that we analysed one cohort of students, disregarding intra-individual variances. Nevertheless, to our best knowledge, we are the first to report situational motivation towards different teaching formats, assessed longitudinally over a period of time, focusing on the affective dimension of learning.^8^ Our results also have particular value in times of the global Covid-19 pandemic, which led to the closure of universities and forced curriculum adaptations, like digital teaching and disruption of SBME.^44^^, ^^45^Medical educators fear the cease of student motivation due to these curriculum adaptations. Our results are reassuring because we showed that classical teachings, like seminars, have similar effects on student motivation. Therefore, even during the pandemic, the affective dimension of learning can be addressed.

## Conclusions

Third-year students reported similar levels of different motivational qualities towards participation in classical seminars and SBME. SBME might not have enhancing effects on student motivation. Therefore, regarding the affective dimension of learning and considering costs and benefits, medical curricula should apply SBME reasonable, not only focusing on SBME as a fashionable and contemporary teaching format. Further research is needed to clarify how the theoretically predicted enhancing effects of SBME on autonomous motivation can be achieved.  During the pandemic, we won't have to fear a decrease in student motivation due to a replacement of SBME with digital teachings. Therefore, we should put maximum effort into creating suitable digital teaching formats, like seminars, as they have proven to have the same effect on the affective dimension of learning.

### Acknowledgements

The authors would like to thank all medical teachers from the Department of Anaesthesiology, University Medical Center Hamburg-Eppendorf.

### Conflict of Interest

The authors declare that they have no conflict of interest.
